# A data plane security model of SR-BE/TE based on zero-trust architecture

**DOI:** 10.1038/s41598-022-24342-y

**Published:** 2022-11-29

**Authors:** Liang Wang, Hailong Ma, Ziyong Li, Jinchuan Pei, Tao Hu, Jin Zhang

**Affiliations:** 1 National Digital Switching System Engineering and Technology Research Center, PLA Strategic Support Force Information Engineering University, Zhengzhou, 450003 China; 2 Network Communication and Security Purple Mountain Laboratory, Nanjing, 210000 China

**Keywords:** Computer science, Information technology

## Abstract

Facing the untrusted threats of network elements and PKI/CA faced by SR-BE/TE (Segment Routing-BE/TE) data plane in the zero-trust network environment, firstly, this paper refines it into eight specific security issues. Secondly, an SR-BE/TE data plane security model ZbSR (ZTA-based SR) based on zero-trust architecture is proposed, which reconstructs the original SR control plane into a "trust-agent" two-layer plane based on 4 components of the controller, agent, cryptographic center and information base. On one hand, we distinguish between the two segment list generation modes and proposes corresponding data exchange security algorithms, by introducing north–south security verification based on identity authentication, trust evaluation, and key agreement before the terminal device establishes an east–west access connection, so reliable data exchange between terminal devices can be realized. On the other hand, for the network audit lacking SR-BE/TE, a network audit security algorithm based on solid authentication is proposed. By auditing the fields, behaviors, loops, labels, paths, and SIDs of messages, threats such as stream path tampering, SID tampering, DoS attacks, and loop attacks can be effectively detected. Finally, through the simulation test, the proposed model can provide security protection for the SR data plane with a 19.3% average incremental delay overhead for various threat scenarios.

## Introduction

Multiprotocol Label switching (MPLS), proposed by Internet Engineering Task Force (IETF), is a new data exchange standard for efficient transmission of data guided by labels on open communication networks. The essence of MPLS is to use label distribution technology. Mapping IP addresses into simple, fixed-length labels and using the labels to forward data has been widely deployed on wide area networks. However, the control plane of MPLS relies on complex Label Distribution Protocol (LDP) and Resource Reservation Protocol (RSVP) protocols, which leads to poor scalability and difficulty in deployment and maintenance.

Therefore, Segment Routing (SR) was born out of MPLS, and it revolutionized MPLS by deleting LDP and RSVP label distribution protocols and adding source routing features, which significantly improved the simplicity of network co24ntrol and the ability of super-large-scale networking^1^. Because of its stateless, easy deployment, cross-domain, and other excellent features, SR fully embodies the new network development concept of "application-driven network". Now, it has been supported by OpenDaylight open source SDN controller and Linux system, which can strongly support the end-to-end traffic scheduling of IP network and programmable reconfiguration of the software-defined network^2^ and become the key technology of SDN/NFV(Software Defined Network/Network Functions Virtualization) in the next step^3^.

SR can be divided into SR-MPLS and SRv6^4^ according to the data plane encapsulation method, and can also be divided into SR-BE(SR Best Effort) and SR-TE(SR Traffic Engineering) according to the implementation mode, in which the SR-BE mode determines the Segment list by the head node through the IGP(Interior Gateway Protocol) shortest path; in SR-TE mode, the SDN controller or SR PCE sends the Segment list to the head-end node through PCEP(Path Computation Element Communication Protocol), BGP(Border Gateway Protocol), BGP-LS, XML, and NETCONF, or the head-end node can automatically generate the Segment list through ODN(On-Demand Next-hop) mechanism, or the operator can explicitly configure it through CLI, NETCONF, etc. The source routing characteristics of SR enable it to specify the key nodes of the traffic path at the head node, and guide the traffic through any path based on the Segment ID (SID), which achieves a delicate balance between control granularity and control simplicity, but also brings new available conditions for attackers to accurately attack the specified links or devices in the domain. However, at present, the academic circles focus on the functional research of SR, such as principle analysis^5^, protocol expansion^6^, technology implementation^7^, and system integration^8^, the research on its security is insufficient, especially the systematic solutions to the threats such as message forgery, identity fraud and node failure faced by its data plane is less.

What aggravates the threat faced by SR is that the network environment is also accelerating the transition to weak trust and zero trust, and Zero-Trust Architecture (ZTA) emerges at the historic moment. This architecture focuses on replacing the default trust granted by traditional network boundary security models (such as firewall, NAT, VPN) through dynamic trust based on multi-factor authentication and fine-grained authorization, to change the security boundary form between the host and the object from fixed hardware to software definition, to fundamentally solve zero trust threats. The progressive nature of ZTA is mainly reflected in the following aspects: the network boundary security models grant long-term trust based on single verification, lacks the traffic inspection inside the boundary, and is challenging to resist threats such as traffic eavesdropping and loopback attacks; while ZTA grants temporary trust based on each verification, which changes the paradigm of trust granting, and implements the security policy of "binding users and devices as network agents, authenticating and granting trust based on network agents, and dynamically authorizing according to trust"^9^, replacing fixed boundaries with dynamic identities, and blocking the lateral movement of attackers^10^.

Focusing on providing the SR-BE/TE data plane security scheme for the zero-trust network environment, this paper applies ZTA to SR-BE/TE and proposes a data plane security model of SR-BE/TE based on ZTA: ZbSR (ZTA-based SR), which focuses on the security of data plane switching device. In this model, the original SR control plane is transformed into a trust plane and an agent plane based on four security components: controller, key center, agent, and information base. Aiming at the two untrusted functions of data exchange and network audit of SR-BE/TE data plane, two list acquisition modes, Segment list generated by switching device in SR-BE/TE and list issued by controller in SR-TE, are distinguished, and the corresponding data exchange security algorithms based on trust evaluation are proposed respectively, that is, before the data exchange in east–west direction data plane terminal device via routing device of SR-BE/TE, in the north–south direction, firstly, it carries out identity authentication based on device information comparison, trust evaluation based on recommendation trust reasoning, and key negotiation based on encryption and digital signature to support it to establish a trusted connection; besides, this paper proposes a network audit security algorithm based on strong authentication, which can detect the attack representations of different threats by auditing the fields, behaviors, loops, labels, paths and SID information of the messages in various directions. Through simulation test and analysis, the proposed model is helpful to deal with different threats faced by SR-BE/TE data plane.

In summary, the main contributions of this article are as follows:

1. 8 kinds of SR-BE/TE data plane security problems facing zero-trust network environment are put forward, and the technical combination basis of SR and ZTA basic function models is summarized;
2. A security model of SR-BE/TE data plane based on ZTA is designed and implemented. For the untrusted function of the SR data plane, two security algorithms of data exchange and network audit are proposed, and 4 sub-algorithms of identity authentication, trust evaluation, key agreement, and loop audit are proposed;
3. The effectiveness of the proposed architecture is proved by cost analysis and simulation, and the shortcomings of high cost were also found.

This paper mainly consists of 5 sections, among which, the second section summarizes and puts forward SR native security mechanism, primary routing security mechanism, SR-BE/TE data plane security problem for the zero-trust network environment, and basic function models of SR and ZTA. The third section expounds the architecture design, component functions, security algorithms, security overhead, and so on of the ZbSR model. In the fourth section, based on the EVE-NG simulation environment, the security performance and overhead of the ZbSR model and the other SR/SDN function models are compared and tested. The fifth section summarizes the full text and looks forward to the following research.

## Related works

At present, as there is no molding solution to the threats faced by SR in the zero-trust network environment, this section mainly summarizes 7 kinds of SR native security mechanisms and 6 kinds of existing mainstream routing security mechanisms, puts forward 8 kinds of SR-BE/TE data plane security problems for the zero-trust network environment, and analyzes the coupling basis of SR and ZTA basic function models.

### SR native security mechanism and primary routing security mechanism

Literature^2,11^ points out the native security mechanisms adopted by SR, summarized into 7 categories in this paper. As shown in Table 1, these security mechanisms can't cope with Zero-trust security threats such as control plane message tampering, denial of service attack, topology based on devices in the domain, and label detection.

**Table 1:** SR native security mechanism.

security mechanism	Implementation method	Threat against
Source routing^2,11^	The head node of the flow encapsulates the label stack to specify the flow path	Malicious drainage
Trust domain^2,11^	Only the source route is used in the domain, and the source route information is cleared by setting the C-flag flag in SRH when the data packet leaves the domain	Label leakage
Package validation^2,11^	RFC8754 stipulates that the optional TLV (Type-Length-Value) object field of SRH in SRv6 message carries HMAC TLV	SRv6 data message tampering
Load leveling^2,11^	Anycast-SID will balance the traffic from a single node to multiple nodes	Single point failure
Fault detect^2,11^	Local trigger (such as BFD(Bidirectional Forwarding Detection)), remote intra-domain trigger (IGP flooding), remote cross-domain trigger (updated by BGP-LS), end-to-end SR Policy survivability detection, explicit candidate path verification and dynamic candidate path recalculation	–
Failure recovery^2,11^	TI-LFA (Topology-Independent Loop-free Alternate) node protection	–
Service hiding^2,11^	Use the “mpls ip-ttl-propagation disable” command to hide the multi-hop MPLS network as a single-hop network, thus invalidating the traceroute command	Traditional topology detection, inter-domain topology detection
	By binding the SR Policy of the specified domain to BSID, users outside the domain cannot obtain the topology within the domain based on the candidate path information	

Literature^12–22^ puts forward a variety of mainstream routing security mechanisms, summarized into 6 categories in this paper, as shown in Table 2. These mechanisms did not consider the label and source routing characteristics of the SR network, and could not directly migrate to the SR scene, nor did they consider and deal with the threats they faced as a whole, so their universality was limited.

**Table 2:** Main routing security mechanisms.

Security mechanism	Examples
Identification inspection	StackPi algorithm for judging the security of forwarding path based on check stack identification^12^; SNAPP algorithm for verification by adding message integrity verification code (MIC) at sender and intermediate node^13^
Node verification	The ICING mechanism checks the received data packets by deploying authentication servers in each node of the network, but it brings high transmission overhead^14^; OSP algorithm grants a certificate between the source and the router, and the intermediate node verifies the data packet according to the certificate, which improves the inspection efficiency but increases the management overhead^15^. RPKI uses digital signature and certificate to authenticate routing source, which can effectively prevent route hijacking^16^; due to the limited deployment of RPKI infrastructure, Tomas and others put forward DISCO, which is based on distributed trust architecture to authenticate routing^17^
Trusted hardware	TrueNet mechanism deploys TCB(Trusted Computing Base) in each node of the network, and determines malicious links through multi-node security information negotiation^18^
Centralization of control	SDN architecture is usually adopted, such as VeriDP algorithm, which verifies whether the data is transmitted normally through control plane policy, thus improving the accuracy of network behavior detection^19^. DFL mechanism collects the verification information of nodes in the transmission path in a centralized way, but it is difficult to avoid a single point of failure^20^
Collaborative filtering	RISP uses RPKI to protect the inter-domain communication of source address, and completes traffic filtering through the cooperation of server, alliance center and AS border router^21^
New technology	Using blockchain to build a distributed trust framework can be used for inter-domain routing protocol to realize IP address prefix authentication^22^

The comparison between the above scheme and the scheme proposed in this paper is shown in Table 3.

**Table 3:** Comparison of 3 types of security solutions.

	SR native security solution	Main routing security scheme	ZbSR security solution
Means	Source routing, trust domain, packet authentication, load balancing, fault detection, fault recovery, service hiding	Identity verification, node verification, trusted hardware, centralized control, collaborative filtering, new technologies	Introduce security component based on ZTA concept
Advantages	Helps to improve security autonomously without additional security mechanisms	Provide adaptive security solutions for a variety of specified network scenarios	Design for segmented routing; Provide comprehensive protection; It can be used in new zero-trust application scenarios
Disadvantages	The source routing feature of the segmented route has security vulnerabilities, which makes it difficult to face some new zero-trust security threat scenarios	Features such as source route and segment label of a segmented routing network are not combined. Lack of comprehensive means of protection	The existing SDP architecture needs to be improved for segmented routing. You can control only the terminal devices that access the domain, but cannot directly control the intra-zone routing devices

### SR-BE/TE data plane security for zero-trust network environment

Based on the above analysis and the premise that "no user, device or application are trusted in the zero-trust environment", this paper defines the SR-BE/TE data plane security problem in the zero-trust network environment as threats of untrusted network element and PKI/CA, which is divided into 8 categories, as shown in Table 4. It can be seen that these problems can be attributed to the unreliable data exchange and network audit function of the SR-BE/TE data plane and the lack of relevant security mechanisms.

**Table 4:** SR security issues for zero-trust network environment.

Security issue		Specific description	Major threats
Untrusted network element	Eavesdropping and replay^23,24^	After the tag of source node and data packet is obtained by eavesdropping, the explicit path of downstream traffic of eavesdropping point will be obtained directly, and replay attack can be realized by replaying normal identity messages to access the network	Confidentiality and Authenticability
	Message Forgery^25,26^	SR is usually only verified at domain boundary devices. By means of routing protocol flooding mechanism, forging control plane protocol messages, or modifying packet headers, it can change critical flow paths or occupy specific link bandwidth, create routing loops, drop traffic, intentionally report errors and other consequences, and destroy link load balance or block network communication	Integrity
	Denial of service attack^2^	According to the SR protocol, when the "segment left" field is non-zero, the router in the domain needs to send ICMP messages to the source address of the data packet. Attackers can use this to force SR nodes to generate and send a large number of ICMP messages, thus realizing DoS/DDoS attacks	Usability
	Identity Deception^27^	Because all the nodes in the SR trust domain are under the unified control, it is usually impossible to implement identity deception in the domain, but the nodes outside the domain may access the SR network as nodes in the domain	Confidentiality and controllability
	Intra-domain detection based on back door of device^27^	By detecting and using the back door of network device, the tag information and data packet payload generated by control protocols such as OSPF for SR are obtained by grabbing packets or tampering with forwarding table entries, and the MPLS/IPv6 tag stack information in them is analyzed to obtain the node tags, link tags and topological relations of downstream device	Controllability and confidentiality
	In-domain detection based on social engineering attack^27^	Log in to the device in SR domain without credit by means of social engineering such as cheating passwords, obtain the label and topology information stored by the device by means of show command, CLI (Command-Line Interface), SNMP (Simple Network Management Protocol) and NETCONF (Network Configuration Protocol) with the help of device maintenance and management tools, and use the device as a sniffing springboard and OAM functions such as MPLS tracert and MPLS ping of SR-MPLS network. Construct attack messages with different label stacks and specific TTL to detect network topology, nodes and links hop by hop. In the network which multi-source manufacturers' devices using different control standards coexist, springboard detection is easier to succeed	Controllability and confidentiality
	Failure of intra-domain node^28^	Due to the lack of stable label release mechanism, modifying SRGB (SR Global Block) of SR router, assigning used labels to it or configuring MPLS label range will lead to service interruption, and the device needs to be restarted	Usability
PKI/CA failure	Failure of infrastructure^29^	Attackers captured network security infrastructure such as PKI/CA (Public Key Infrastructure/Certificate Authority) through APT attacks, which led to the failure of traffic encryption in SR domain	Confidentiality and Authenticability

### Coupling foundation of SR and ZTA basic function model

**Figure 1:**
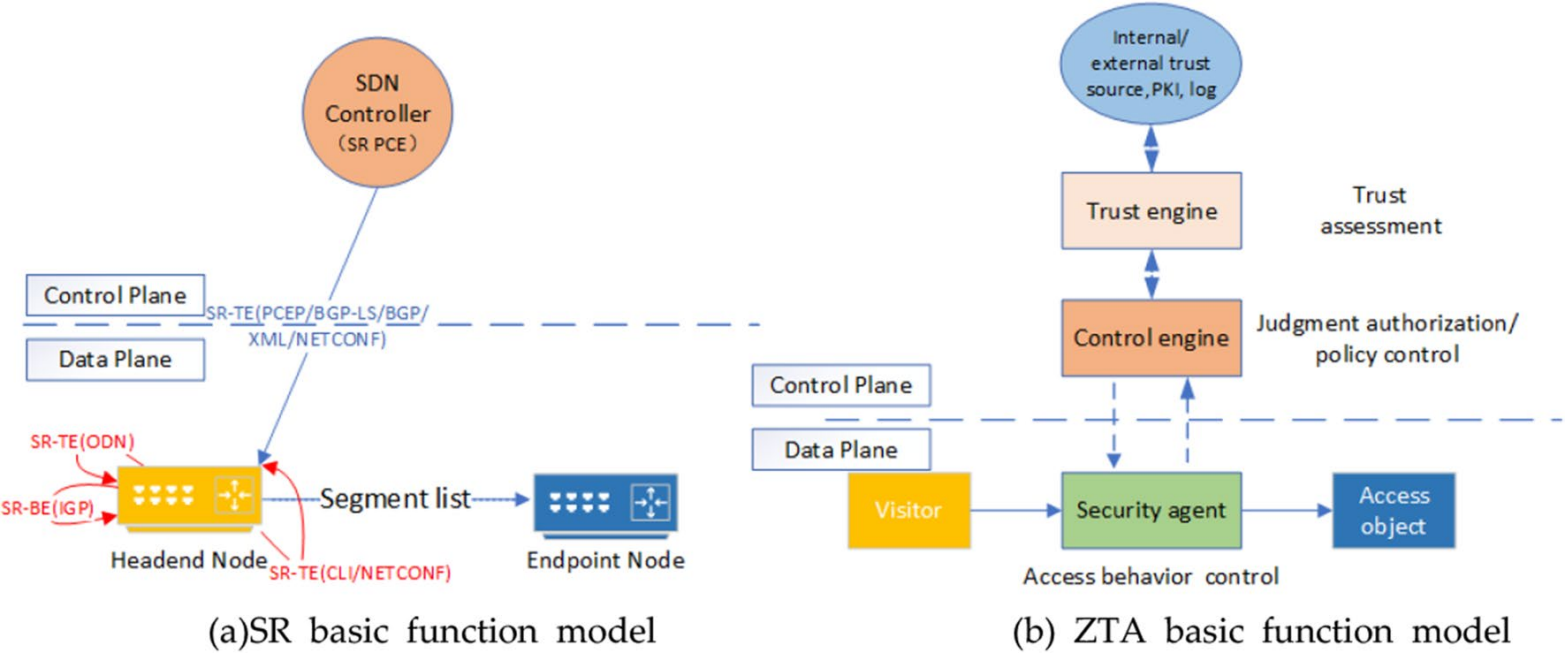
SR basic function model and ZTA basic function model.

Figure 1a,b are the basic functional models of SR and ZTA, respectively, in which components with similar functions are identified with the same color. As shown by the red line and blue line in Fig. 1a, there are two generation modes of Segment list in SR basic function model: self-generation of network element and issuance of the controller; SDN controller, as its control engine, lacks the trust engine for managing trust and internal and external information sources, PKI, logs and other components for storing identity in ZTA model of Fig. 1b, which leads to its unreliable data exchange and network audit functions. Therefore, the two functional models have a certain coupling foundation, and the ZTA trust engine can be integrated into the SDN controller.

### The previous work

In the early stage, we mainly have a related research result on SR data plane security model, and the difference between it and this work is shown in Table 5.

**Table 5:** Comparison between previous work and this work.

	SbSR (SDP based SR)^30^	ZbSR (ZTA based SR)
Problem oriented	The terminal device of the SR network data plane	The switching device of the SR network data plane
Modeling	The migration model of the mature SDP model is carried out	Based on the concept of ZTA, a new ZTA model is designed by adding security components and reassembling the original functional components
Assessment	Port scanning; Traffic monitoring; DoS attack; Topology detection based on label detection; Routing loop attack based on directional label; Performance overhead	Control plane message tampering; Data plane loop attack; Identity deception; Back door utilization; DOS attack; Performance overhead

## SR-BE/TE security model (ZbSR) based on ZTA

**Figure 2:**
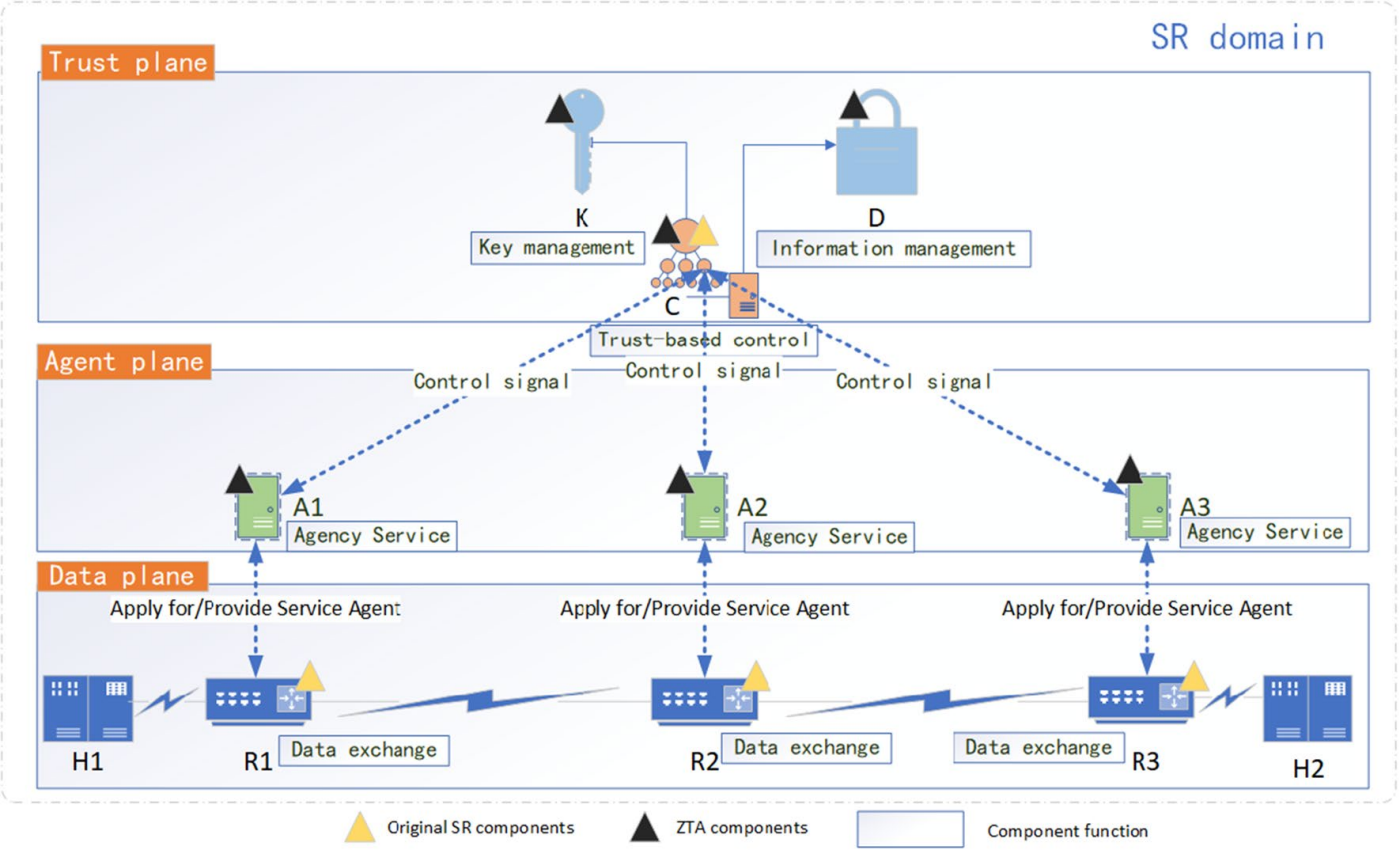
ZbSR security model.

Based on the above analysis, the ZbSR security model proposed in this paper is mainly composed of trust plane, agent plane, and data plane, as shown in Fig. 2, in which the trust plane is composed of the controller (C), key center (K) and information base (D), which is connected to the data plane through agent plane, and whitelist access control is established between planes, which is responsible for centralized control, authentication and trust calculation of data plane devices, in which the controller is based on the expansion of the original SDN controller of SR architecture. The agent plane consists of agent (A) connected in series to each SR data plane device, responsible for providing security agency services such as encryption, auditing, and reporting. The data plane is composed of switching devices such as SR router (R) and terminal device such as host (H), which is responsible for generating data and transmitting traffic.

The ZSR model is modeled with symbols and definitions in Table 1.

### ZbSR model component function and modeling

The function of the ZbSR model component is that the controller calls the key center (for managing keys) and the information base (for managing and storing identity information) to control the subordinate plane based on trust, and the agent provides security agency services for the data plane.

#### The controller (C)

The controller consists of the original SDN controller and the trust engine expansion module. It is responsible for realizing access control, path delivery, and other functions based on authentication and trust calculation.

1. Segment list issuing

In SR-TE, a path is issued for the data plane by generating a Segment list, the Segment list *SL* is shown in formula (1).

1 $$ SL = \left\{ {SID_{1} ,SID_{2} ,SID_{3} , \ldots ,SID_{i} , \ldots ,SID_{j} , \ldots ,SID_{n} } \right\} $$

(2) Authentication and authorization

Referring to the security design based on identity control access^31,32^ in Software-Defined Perimeter (SDP)^33^, the trust engine of the controller performs identity authentication and trust evaluation on the devices in the domain, and then implements the minimum authorization^34^, and then the authentication and authorization results are handed over to the SDN controller, which issues control signaling. The access subject and object 5-tuple authorization information Credit is modeled as shown in formula (2), where *Smac* and *Dmac* represent the MAC addresses of the access device and the visited device respectively, *SID* and *pSID* represent the Prefix Node SID assigned by the access device and the visited device respectively, and *P* represents the access protocol.

2 $$ {\text{Cre}}dit\mathop  = \limits^{{def}} \{ {\text{Smac}} + Dm{\text{ac  +  }}SID + P + pSID\}   $$

According to the ZTA concept, the authorization mode can be divided into centralized authorization and separate authorization. Centralized authorization means that after authentication and trust evaluation are carried out on the network-connected devices, the list of accessible devices and protocols is granted in a centralized way in the form of an authorization list. As shown in formula (3), the authorization list contains 6 types of information, among which, *D*^*i*^, *Cert*^*i*^, *t*_*Certi*_, *PK*_*Di*_, *P* and *K*_*D*_(*i*) respectively represent the accessible device i, the access certificate of device I, the lease period of the access certificate of device i, the public key of device i, the access protocol and Separate authorization means that device A needs to verify authorization every time it accesses device B through the new protocol. At this time, the authorization information is shown by formula (5), including the access certificate of device B, the lease period of the access certificate of device B, the public key of device B, the access protocol, and the traffic encryption key. Compared with centralized authorization, separate authorization not only achieves fine-grained control but also brings more overhead. Therefore, this paper sets two authorization modes that can be switched as needed.

3 $$ List_{A} = \left\{ {D,Cert_{A} ,t_{{Cert_{A} }} ,PK,P,K_{D}^{A} } \right\} $$

4 $$ \left\{ {\begin{array}{*{20}l} {D = \left\{ {D^{1} ,D^{2} ,D^{3} , \ldots ,D^{n} } \right\}} \hfill \\ {Cert_{A} = \left\{ {Cert_{A}^{1} ,Cert_{A}^{2} ,Cert_{A}^{3} , \ldots ,Cert_{A}^{n} } \right\}} \hfill \\ {t_{{Cert_{A} }} = \left\{ {t_{{Cert_{A}^{1} }} ,t_{{Cert_{A}^{2} }} ,t_{{Cert_{A}^{3} }} , \ldots ,t_{{Cert_{A}^{n} }} } \right\}} \hfill \\ {PK = \left\{ {PK_{{D^{1} }} ,PK_{{D^{2} }} ,PK_{{D^{3} }} , \ldots ,PK_{{D^{n} }} } \right\}} \hfill \\ {P = \left\{ {P_{{D^{1} }} ,P_{{D^{2} }} ,P_{{D^{3} }} , \ldots ,P_{{D^{n} }} } \right\}} \hfill \\ {K_{D}^{A} = \left\{ {K_{D}^{A} (1),K_{D}^{A} (2),K_{D}^{A} (3), \ldots ,K_{D}^{A} (n)} \right\}} \hfill \\ \end{array} } \right. $$

5 $$ List_{A} (B) = \left\{ {Cert_{A}^{B} ,t_{{Cert_{A}^{B} }} ,PK_{B} ,P_{B} ,K_{D}^{A} (B)} \right\} $$

(3) Rules issuing

Before the SR source node starts streaming according to the Segment list, the controller issues security rules for preventing path tampering to the agents of each node in the list, detailed in Section “Network audit security algorithm based on solid authentication”.

(4) Device control

The controller centrally controls all devices in the domain, centrally configures their Prefix-SID to prevent the attackers from tampering, and timely removes the failed devices from the list of available devices and recycles their SIDs; storing the suspected malicious device behavior found in the detection into the information base, disabling its access credentials and reporting to the network administrator when the negative feedback accumulation causes its trust to be lower than the threshold; provide the central working clock for each component of the system and provide a unified time reference.

(5) Keys scheduling

Through the agent plane of the controller, the key center is called to centrally distribute the traffic encryption key and other keys to the protocol peers that have been authorized successfully.

#### Key center

**Figure 3:**
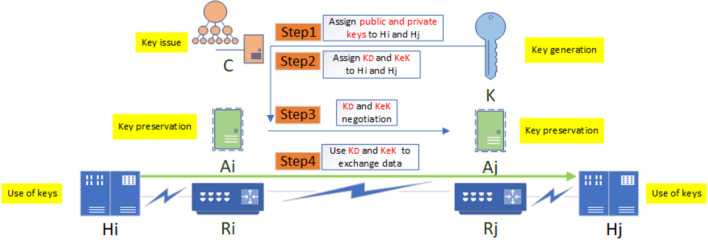
ZbSR key usage process.

The key center is used to centrally control the keys in the domain and prevent the potential safety hazard of key decentralized configuration^35^. It adopts the popular "symmetric password-asymmetric password" mixed encryption mechanism^36^, in which the fast symmetric password is used for traffic encryption/decryption, and the slow asymmetric password is used for key exchange and signature verification; because ZTA doesn't trust public PKI/CA, the key center is used as the private CA in the domain to issue digital certificates to the terminal devices in the domain^37^. The managed keys include traffic encryption key *K*_*D*_, key-encryption key *KeK*, its own public and private keys *K*_*pub*_ and *K*_*pri*_, and the public and private keys *R*_*pub*_ and *R*_*pri*_ of each terminal device. All keys are replaced regularly to prevent abuse. To simplify the configuration, duplicate keys can be configured for the nodes in an SR Anycast group. The use of all kinds of keys can be divided into 4 steps, as shown in Fig. 3.

- Step1 K preallocates the public and private keys for all terminal devices, sends them through C, deposits them in A, and replaces them regularly;
- Step2 K allocates ${K}_{D}$ and *KeK* as needed for data exchange between H_i_ and H_j_, which is issued by C, stored in A, and replaced regularly;
- Step3 A_i_ and A_j_ use the public and private keys of H_i_ and H_j_ to negotiate *K*_*D*_ and *KeK*;
- Step4 H_i_ and H_j_ exchange data with *K*_*D*_ and *KeK*.

#### Information base

The information base is used to store and manage device authentication information and protocol authorization information. The device authentication information is related to authentication information of the device itself, such as username/password, SID, router-mac, etc.,^38^ which is determined by the formula (6), and the protocol authorization information is related to the protocol authorization. Such as *Whitelist* of connection, routing protocol type *P*_*R*_, link Adjacency *Adj*_*ij*_, port number *Interface*, peer IP address *IP*_*p*_, etc., are determined by the formula (7), in which *Adj*_*ij*_ is determined by the adjacency matrix of link, as shown in formula (8), which describes the adjacency of device, with 1 indicating adjacency and 0 indicating non-adjacency; the Whitelist of connections is determined by the formula (9), which specifies all permitted connections in the domain, and the information in the information base is dynamically updated with the change of network devices.

6 $$ I_{a} (i)\mathop = \limits^{def} \{ Uname + Upass + SID + Rmac\} $$

7 $$ P_{a} (i,j)\mathop = \limits^{def} \left\{ {Interface_{i} + Adj(i,j) + Whitelist + P_{R} + IP_{j} } \right\} $$

8 $$ Adjacency = \left( {\begin{array}{*{20}c} {Adj_{11} } & \ldots & {Adj_{1m} } \\ \vdots & {Adj_{ij} } & \vdots \\ {Adj_{m1} } & \cdots & {Adj_{mm} } \\ \end{array} } \right),Adj_{ij} = (1,0) $$

9 $$ Whitelist = \{ (SID_{i} ,SID_{j} ), \ldots ,(SID_{i} ,A_{k} ), \ldots ,(A_{k} ,C), \ldots \} $$

#### Agent

The agent is used to provide security agency service for data plane devices, and it is directly connected with each SR switching device^39^. There is no direct connection channel between agents, to prevent malicious nodes from bypassing trust plane supervision and direct communication. The agent mainly has 4 functions: key management, path report, log record, and behavior audit. Key management means that the agent provides key negotiation agent services for data plane devices; path report implies that after the SR head node generates the flow path, it needs to report the path to the controller through the agent for decision-making; logging refers to recording the behavior log of SR switching device to trace the malicious behavior; behavior audit refers to auditing the behavior of data plane devices together with the controller according to the network audit security algorithm in Section “Network audit security algorithm based on solid authentication”.

### Data exchange and network audit security algorithm of ZbSR model

To ensure the integrity, confidentiality, and availability of data in the SR domain, the ZSR model introduces five security mechanisms: packet authentication, data encryption, check and filtering, security audit, and trust renewal.

#### Data exchange security algorithm based on trust evaluation

**Figure 4:**
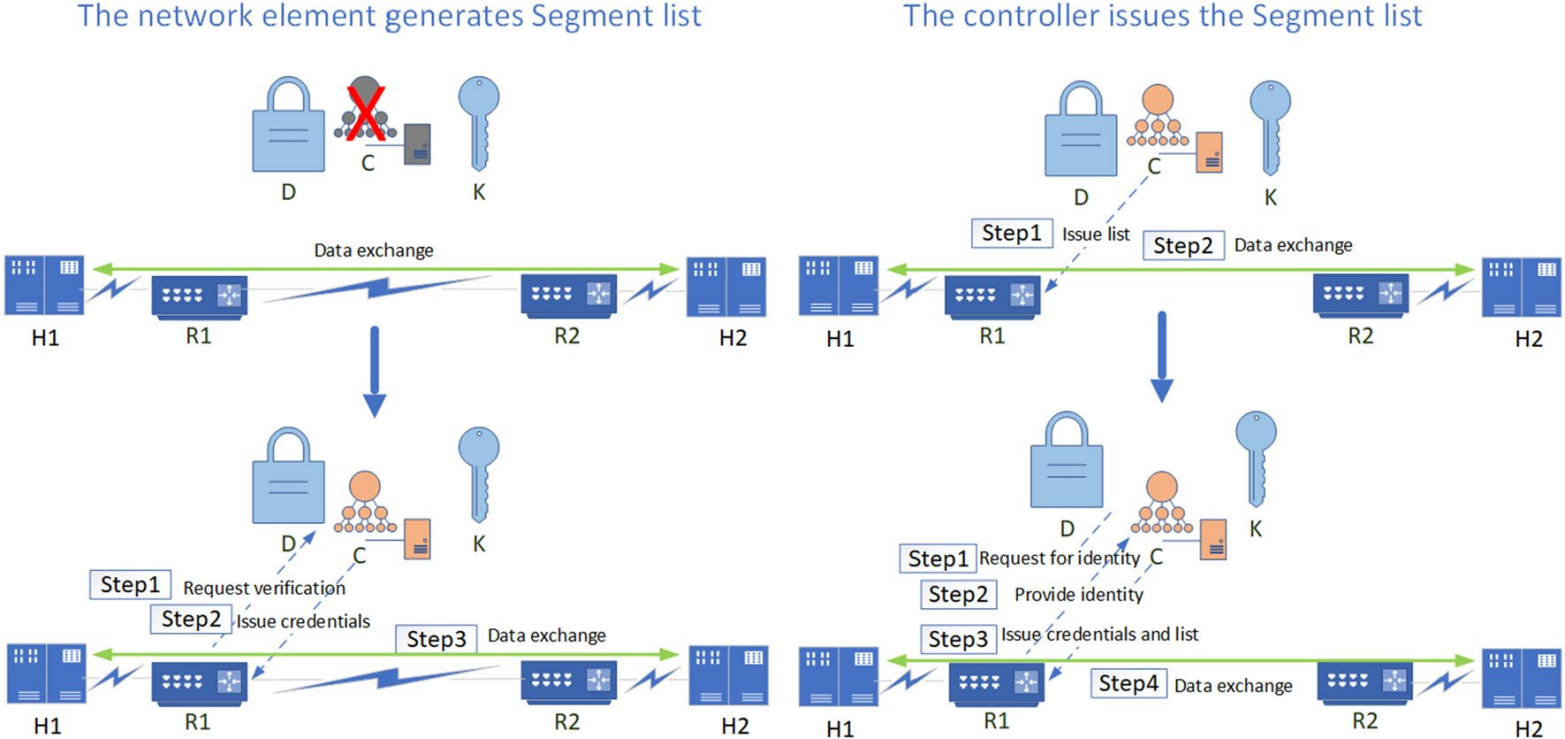
Simplified ZbSR access process.

To realize reliable east–west data exchange between switching devices, firstly, based on ZTA's security design of "first authentication, then connection", a UDP-based SPA (Single Packet Authorization) method is adopted to initiate pre-authentication to the trust plane, and the trust plane carries out the north–south security authentication based on identity authentication, trust evaluation, and key negotiation. Secondly, the terminal device realizes the encrypted traffic exchange by encrypting traffic with a key. Taking the separate authorization mode as an example, the simplified process of terminal H1 accessing H2 is shown in Fig. 4, implemented in two modes: Segment list generation by the network element and Segment list distribution by the controller.

1. Mode for the network element to generate Segment list

**Figure 5:**
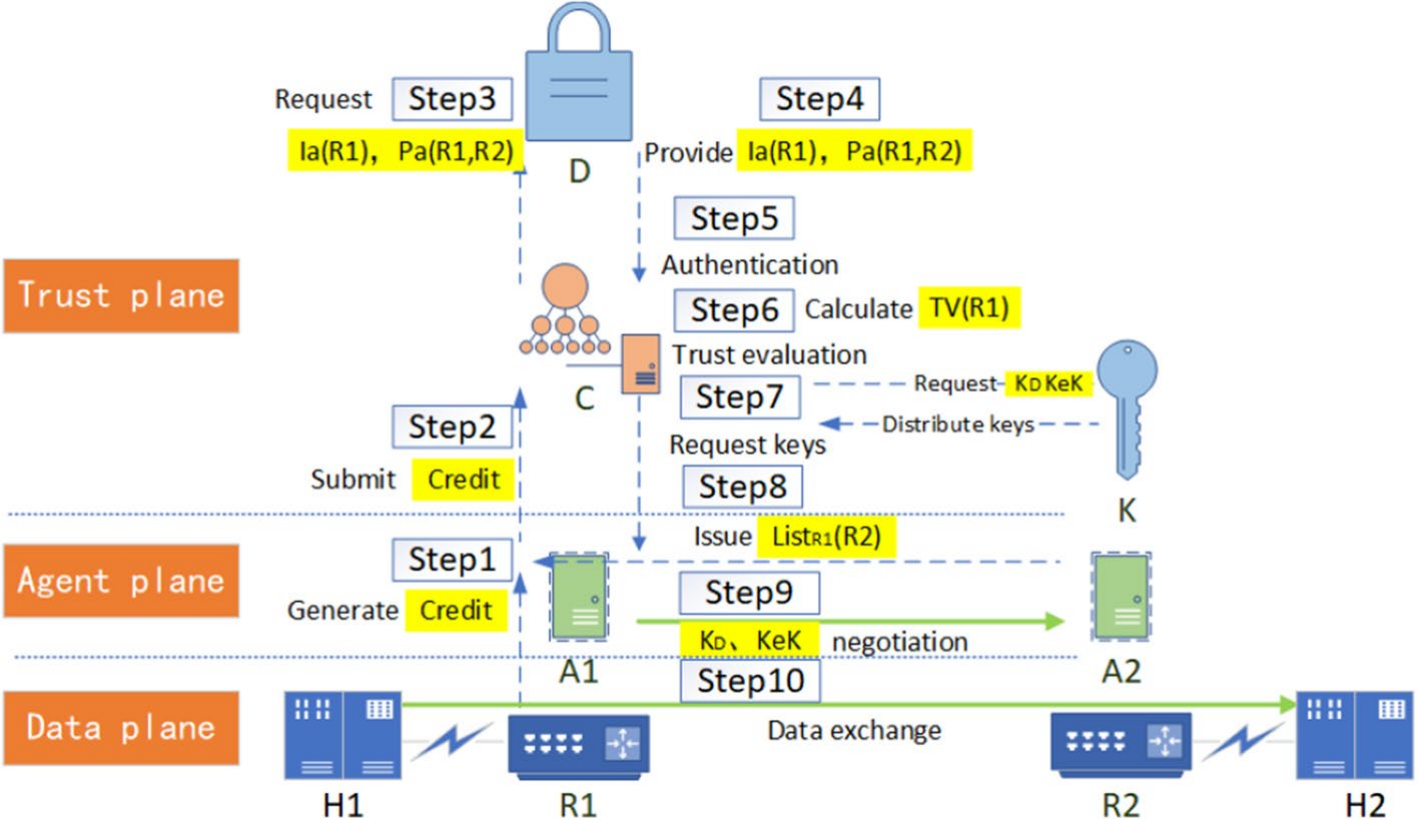
Data exchange process in the mode for the network element to generate Segment list.


In this case, the data exchange process is shown in Fig. 5, and the pseudo-code of the process is shown in Algorithm 1.

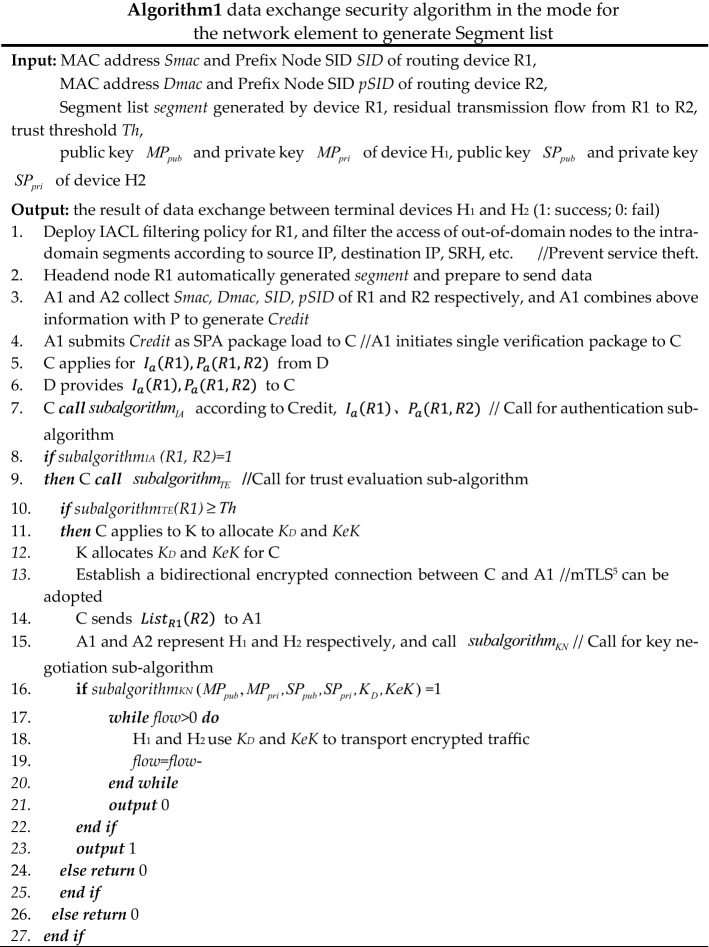

(2) Mode for the controller to issue Segment list

**Figure 6:**
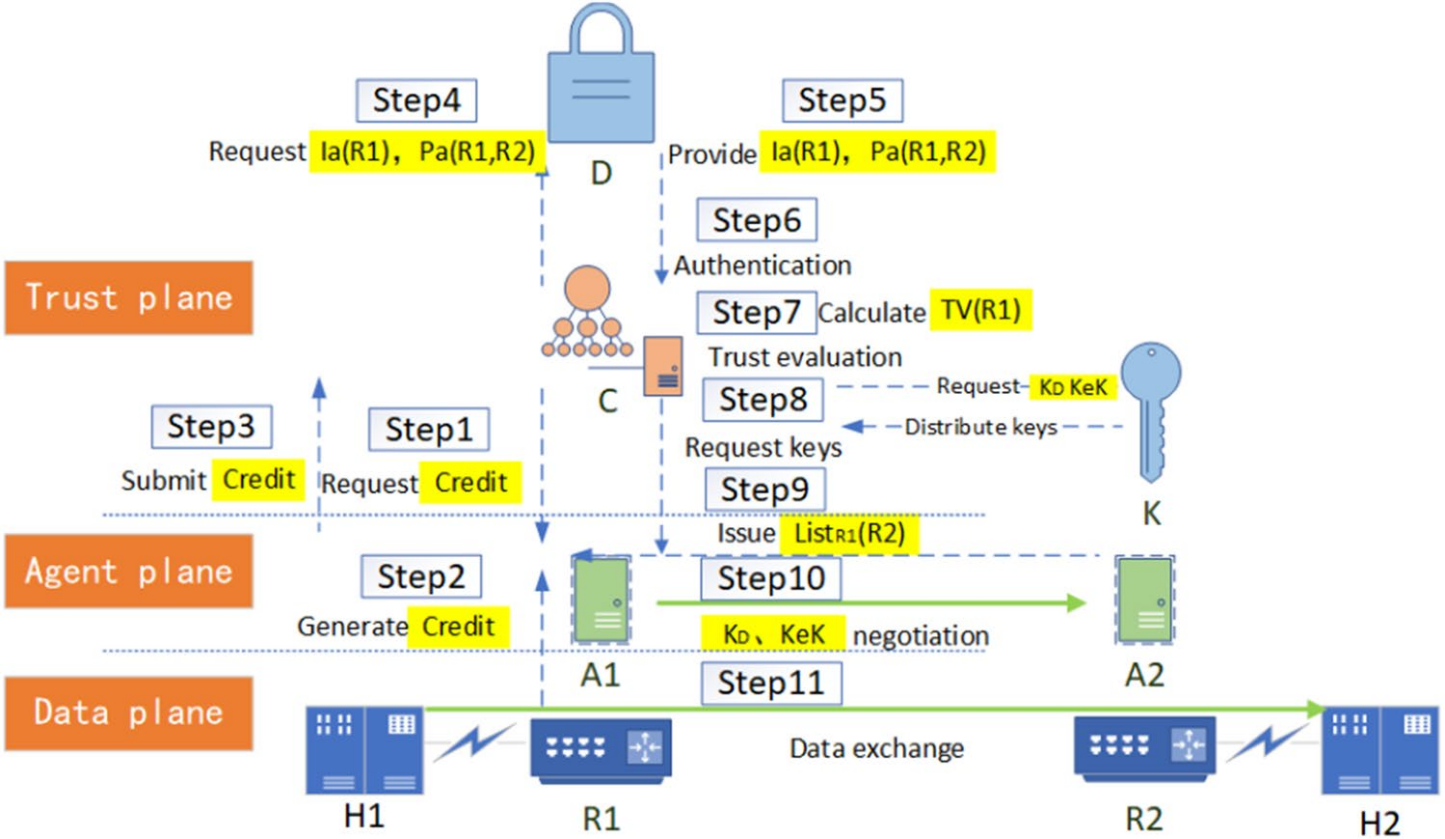
Data exchange process in the mode for the controller to issue Segment list.


In this case, the data exchange process is shown in Fig. 6, and the pseudo-code of the process is shown in Algorithm 2.

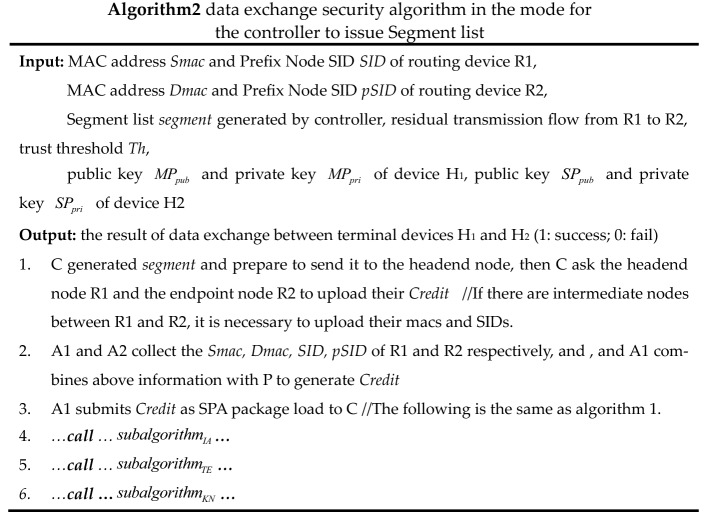


The authentication sub-algorithm, trust evaluation sub-algorithm, and key negotiation sub-algorithm called by algorithms 1 and 2 are shown as *subalgorithm*_*IA*_, *subalgorithm*_*TE*_*,* and *subalgorithm*_*KN*_. In *subalgorithm*_*TE*_, trust renewal can be implemented for temporary trust granted based on security metrics, but this scheme has not been implemented in this paper due to limited research energy.
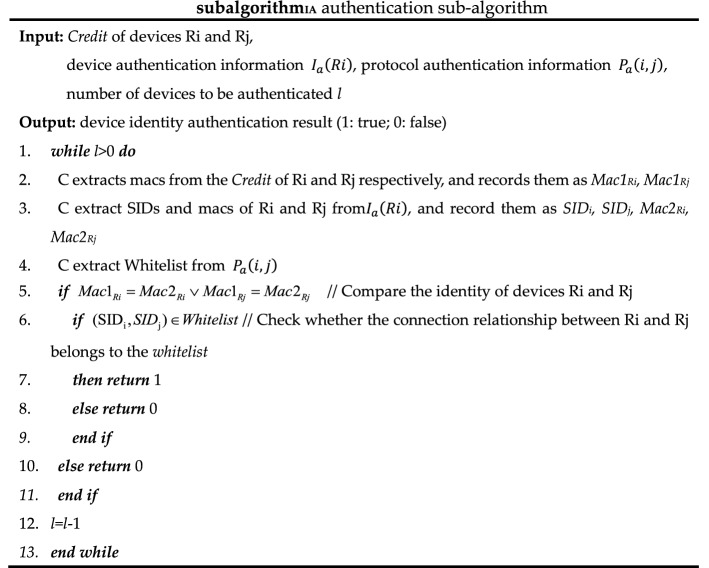



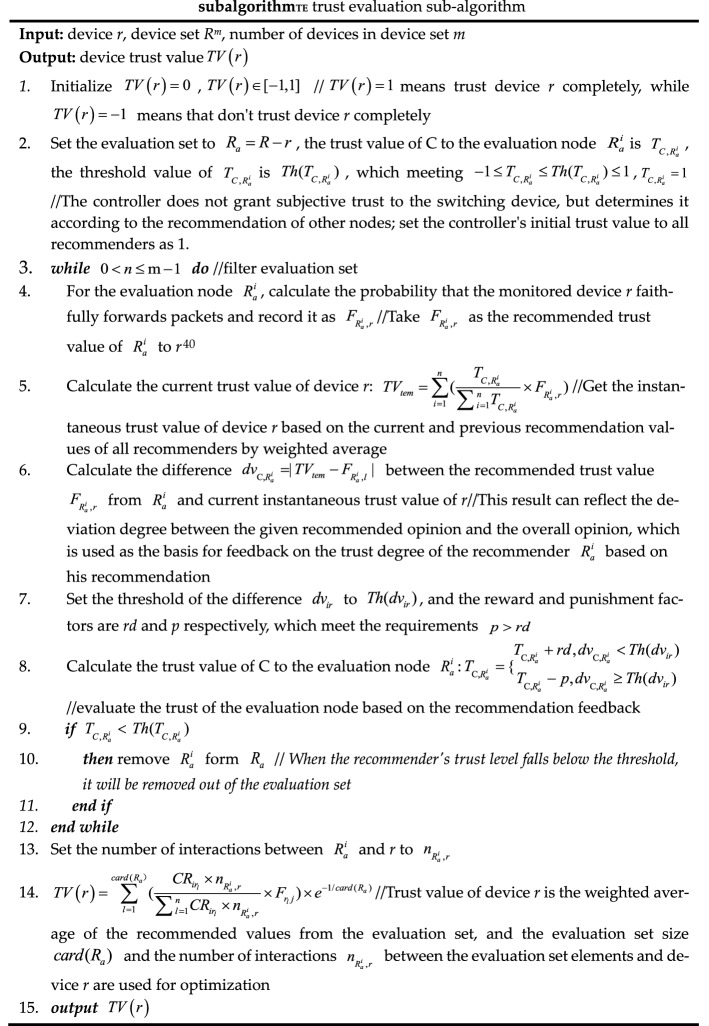



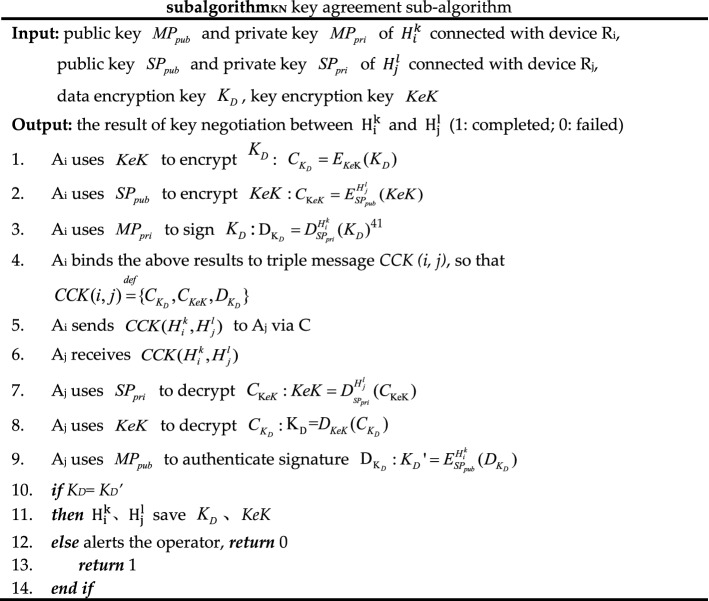


#### Network audit security algorithm based on solid authentication

Due to the lack of audit mechanism for threat representation in SR network, a network security audit algorithm is proposed based on ZTA's strategy of solid verification of all behaviors in the domain. The pseudo-code of related process is shown in algorithm 3. The audit content includes the following 6 aspects.

**Figure 7:**
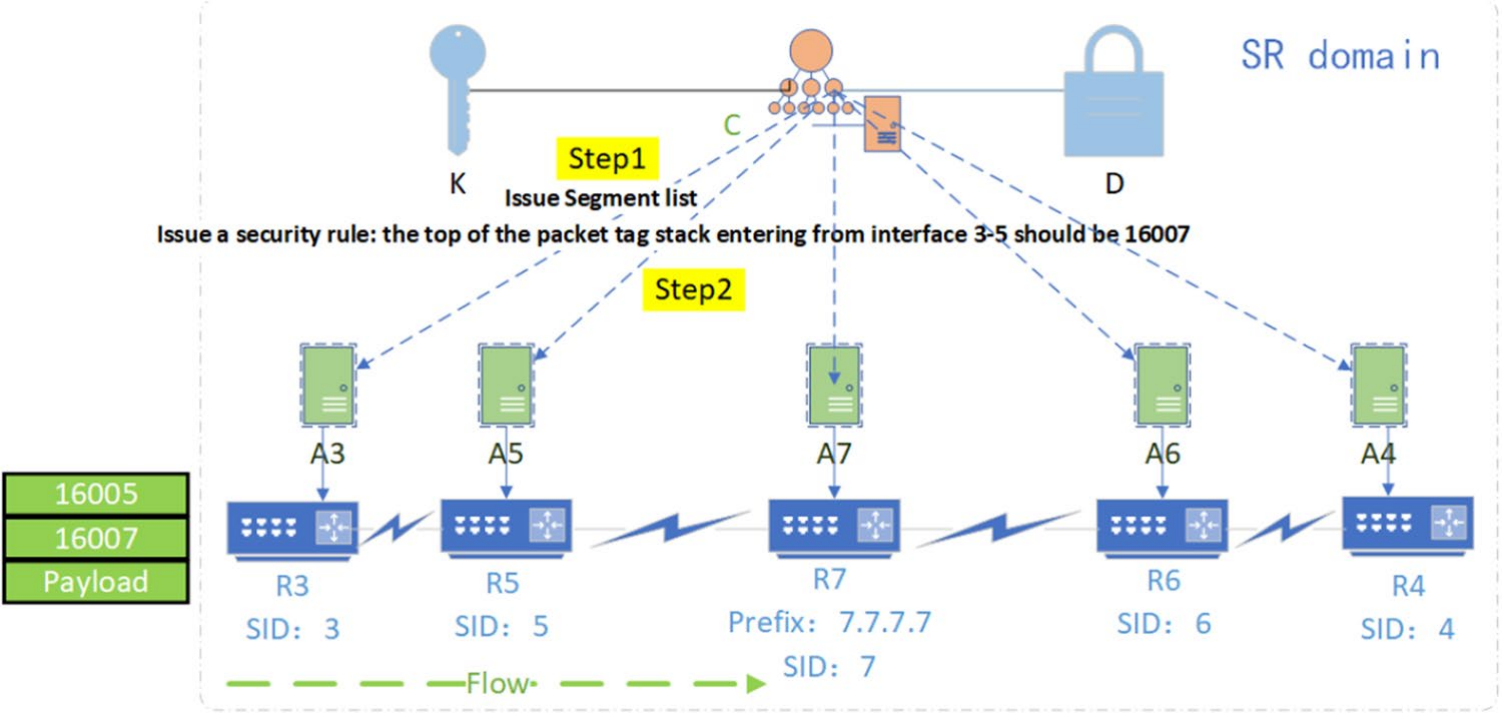
Schematic diagram of SR rule audit.

**Figure 8:**
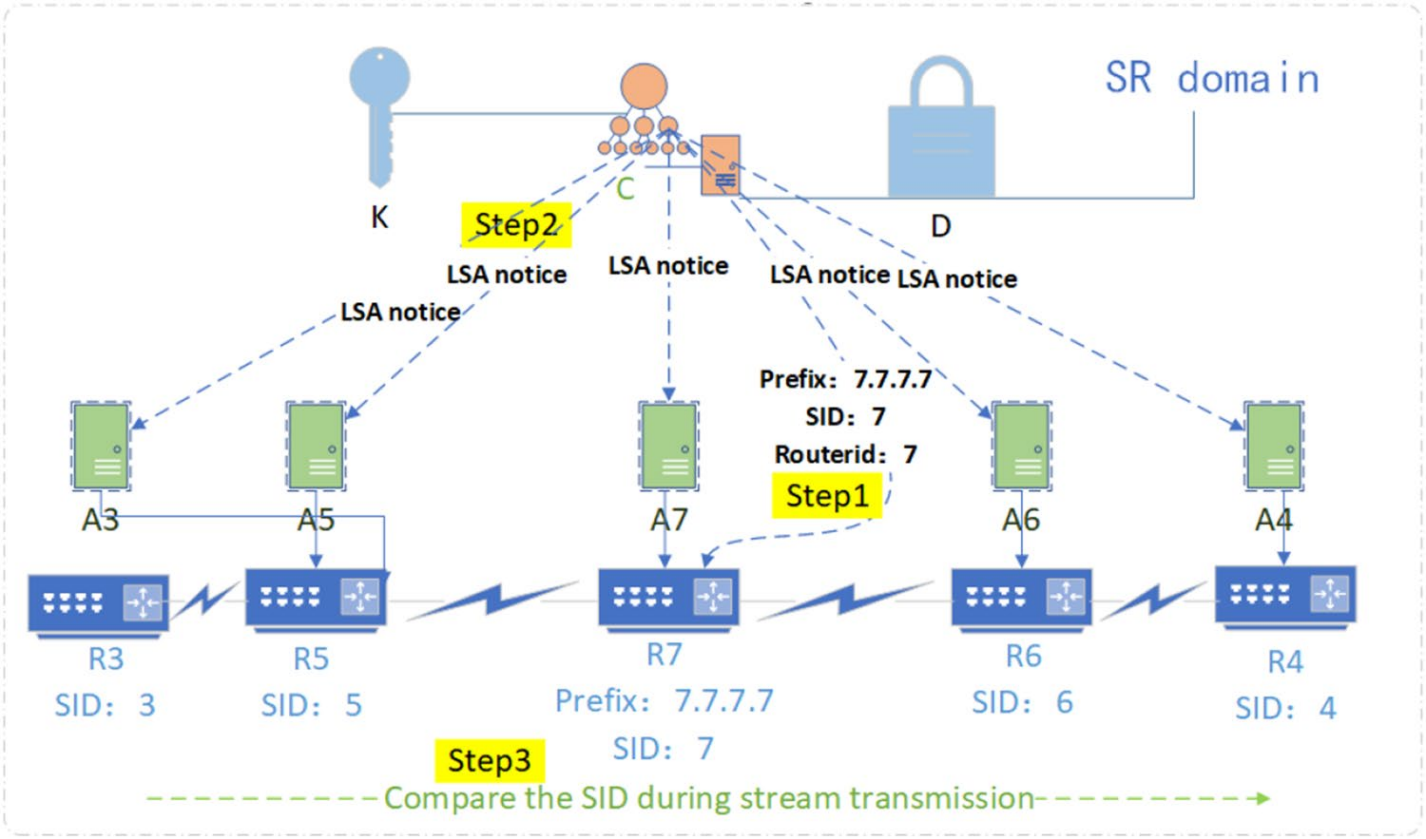
Schematic diagram of SR SID audit.


1. Field audit: audit whether the TTL value of the packet header is legal and whether the outbound traffic of the domain egress router has removed the SRH.
2. Behavior audit: audit whether the rate of ICMP information generation reaches the threshold for enabling the ICMPv6 rate-limiting mechanism and whether the traffic which cannot find next-hop to be malicious.
3. Loop audit: if the label stack only uses Prefix-SID, then directly determine whether there is a loop according to the following subalgorithmLP; if the label stack contains Adjacency-SID, restore the network topology according to the label stack, and then determine the loop according to subalgorithmLP.
4. Label audit: audit the validity of SRGB labels, SRLB labels of specific border routers, and other external labels.
5. Path audit: audit whether the flow path has been tampered with by malicious intermediate nodes. As shown in Fig. 7, the controller issues a segment list {16,007} to node 3, and according to the list, issues security rules to all intermediate nodes (node 5 at this time) along the path: the top label of the received packet from the interface from node 3 to node 5 should be 16,007; otherwise, it is discarded.
6. SID audit: audit whether the SID of a flow path node has been tampered with by malicious intra-domain nodes; it can be divided into two steps. As shown in Fig. 8, the controller centrally configures the Prefix-SID, router-id of each device node, imports them into the information database in advance, and synchronizes them to each device node through LSA notifications. Each device node caches its own and other node SIDs to Label Manager (LM); in the first step, when each device node receives a new LSA notification, it will be audited and compared with the SID cached by the LM. It will be considered valid and received only if the matching is successful. If the matching fails, it will report an exception to the controller, then the controller determines whether there is an attack; the second step is to refer to LM and FIB (Forwarding Information dataBase) to audit whether the SID has been tampered with during streaming. If an unrecognized SID is found in the LM, it will be further matched in the information database. If the matching fails, it will be reported to the operator.

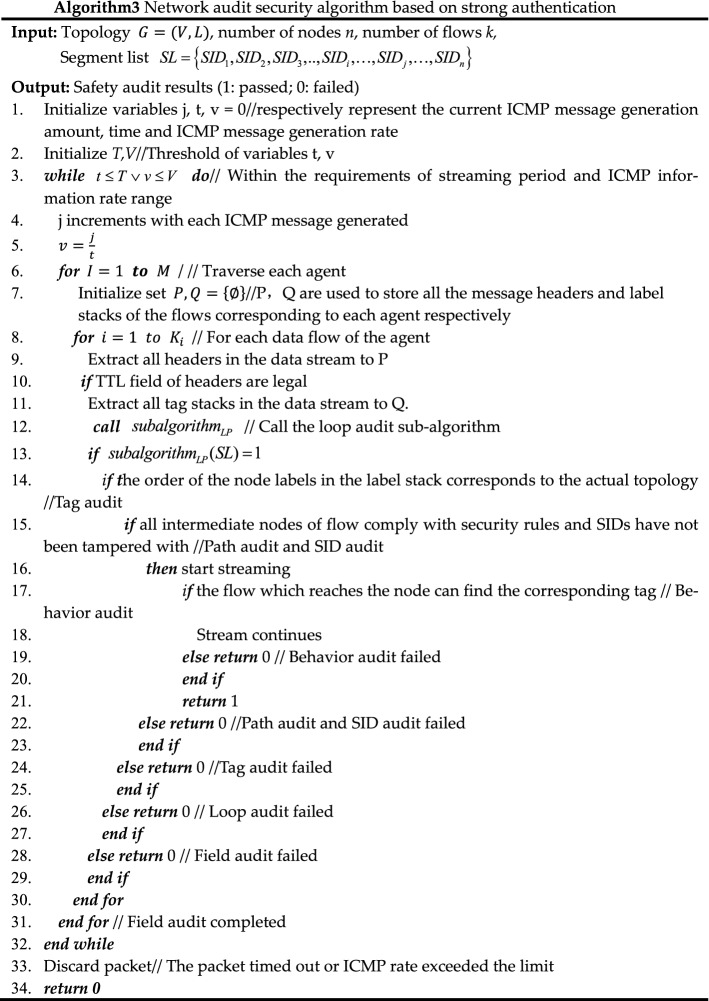


The subalgorithmLP called in algorithm 3 is as follows.


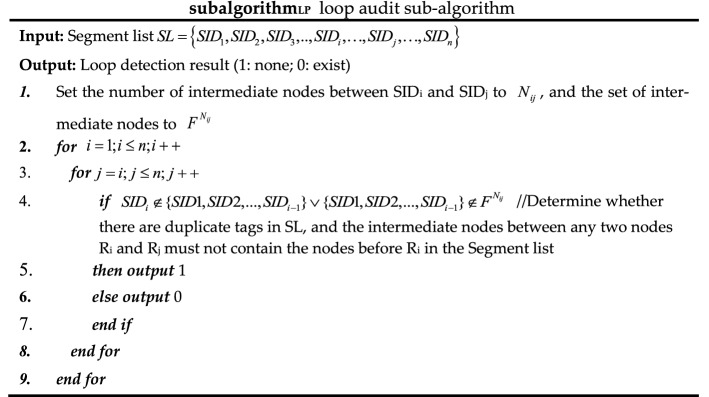


### ZbSR model security overhead

According to different components, the security cost of the ZbSR model is divided into 6 parts, that is, controller cost, key center cost, information base cost, agent cost, encryption cost of terminal devices, and component synchronization cost. Because all kinds of security components run in parallel, in addition to the one-time hardware cost brought by the introduction of devices, the evaluation of system performance cost only needs to pay attention to the time delay item that has the most significant influence on streaming transmission. The related symbols and definitions are shown in Table 6.

**Table 6:** Definitions of symbols in the ZbSR model.

Symbol	Definition
M	Number of agents
${\text{N}}$	Number of device nodes
${\text{N}}_{t}$	Number of nodes in communication
$N_{f}$	Flow quantity
$N_{f}^{i}$	Number of streams that agent i flows through
k1	Flow speed
k2	Constant
$n_{i}$	Number of message segments in each stream
µ	Key update period

The first is the controller overhead. If the performance allows, controller can be deployed single, and it can also be deployed multiple to realize load balancing and disaster recovery. The cost is related to the number of devices N it controls, and the cost is associated with the number of streams ${N}_{f}$ when it issues paths and rules. When authenticating, the cost is related to N; when controlling the device, it is only performed when the device leaves the network, or malicious device is generated, and the occurrence probability is small and can be ignored; when scheduling the key, it is only issued to the nodes in communication after the key is updated, so the computational complexity of the controller overhead is $O(N+{N}_{f})$. The second is the key center overhead. There is only one set in the SR domain, and the cost mainly comes from its regularly key updating, and its computational complexity is $O\left( {N_{t} \times \frac{k2}{\mu }} \right)$. The third is the information base overhead. In-domain devices cache commonly used verification information to the local agent, and the information base only needs to import information when the topology is established, verify information when new users access the network, and update the information when devices change, so the overhead is negligible compared with the controller. The fourth is the agent overhead. The agent is used in every streaming for key management, path reporting, and logging, which is related to ${N}_{f}^{i}$; in behavior audit, the time complexity of field audit is $\sum_{1}^{{N}_{f}}(k1\times {n}_{i})$: Behavior audit is triggered only when abnormal traffic occurs, and the overhead can be ignored. Other auditing functions are only related to the number of streams $N_{f}^{i}$, so the computational complexity of agent overhead is $O\left( {\mathop \sum \limits_{1}^{{N_{f} }} \left( {k1 \times n_{i} } \right) + \sum N_{f}^{i} } \right)$, namely $O\left( {\mathop \sum \limits_{1}^{{N_{f} }} \left( {k1 \times n_{i} } \right) + N_{f} } \right)$. The fifth is the encryption overhead of terminal devices. If hardware devices are used for encryption, the efficiency is high, so the time delay can be ignored. However, if software devices are used for encryption, it will take more time. The sixth is the component synchronization overhead. Usually, there is only one controller deployed in the domain, and the information between agents does not have to be identical, so only the key negotiation and authentication need to be synchronized. Here, the Raft state synchronization technology is implemented according to the flow information between devices, and the overhead is low and can be ignored. It can be seen that the security overhead of the ZbSR model is concentrated in 3 parts: controller, agent, and terminal device encryption. The security cost comparison between this model and other similar routing security models is shown in Table 7. It can be seen that compared with other models, the ZbSR model brings more hardware cost and time cost due to the introduction of new security components and security mechanisms, but this is necessary, and the reasons have been explained in Table 3.

**Table 7:** Security overhead comparison of various security models.

	Time overhead	Storage overhead	Hardware overhead
ZbSR	More time overhead is introduced for the authentication and encryption mechanisms are introduced at the same time	The storage overhead is focused on the newly added security components, so the original data plane devices have no new security overhead	The key center, agent and information base are newly introduced
ICING^14^	More than 10,000 cycles	23.3% more expensive than a standard IP router	93% more expensive than a standard IP router
OSP^15^	Less than 1000 cycles	Lower storage consumption compared to ICING^14^	No assessment
MFRA^42^	9% of the traditional OpenFlow solution	No assessment	No assessment

## Simulation test and analysis

### Simulation settings

**Figure 9:**
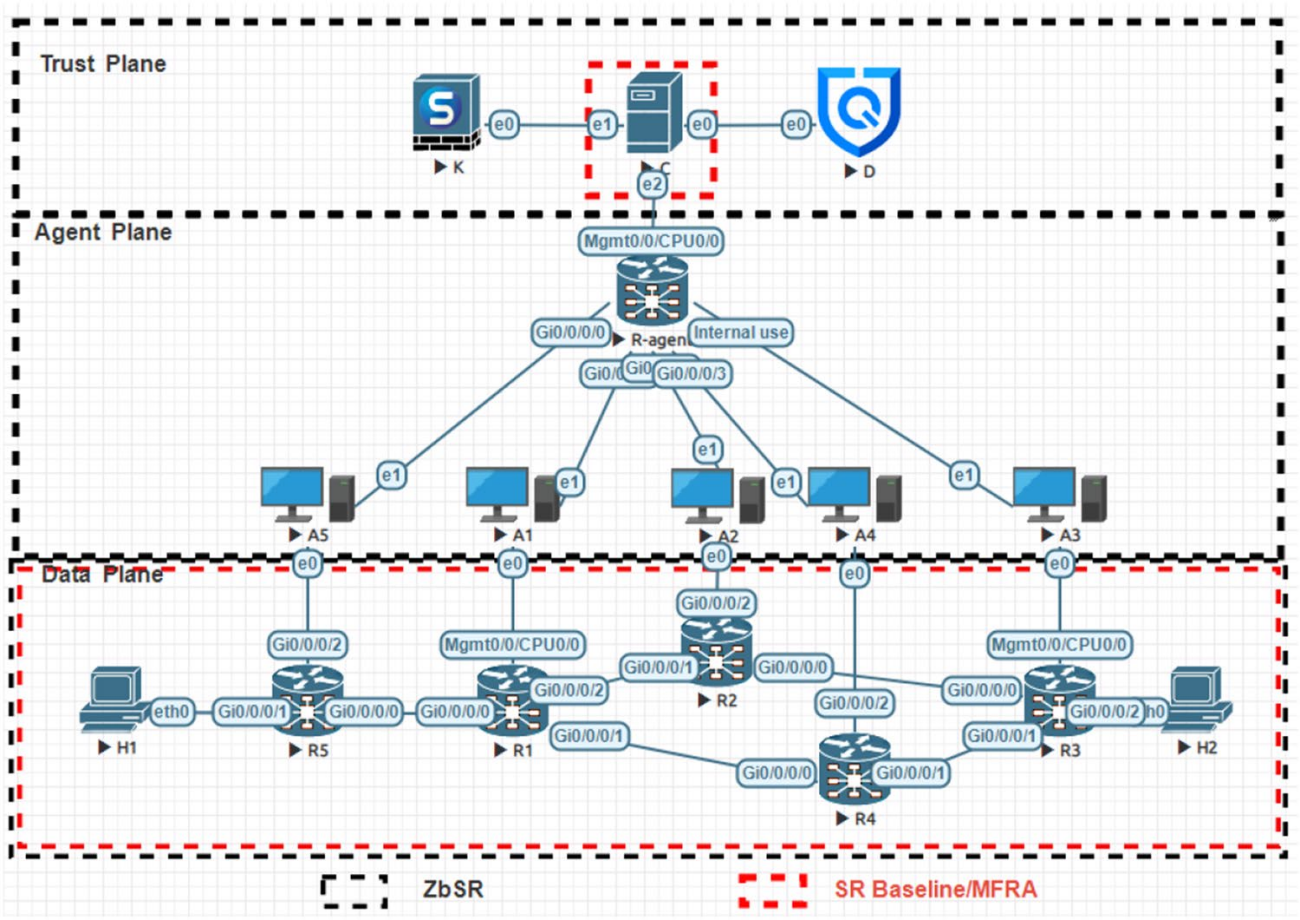
Simulation network topology.

OpenDaylight open-source controller is installed based on KVM virtual machine in EVE-NG 3.0.1-16 PRO, and its function is programmed to realize ZbSR controller. Dedicated Linux virtual machine is used as agent. Because it is challenging to build private CA, information base, and encryption hardware, and it is not the focus of research, this paper adopts a simplified design and uses virtual machines based on X.509 protocol and DES encryption software to simulate key center. Virtual machine simulation information base based on MySQL database. Due to the lack of mature and comparable SR security models, the ZbSR model is compared with the SR Baseline model, the MFRA model, the SDN cross bitmap algorithm model^43^, and the DoS attack detection model based on C4.5^44^, among which the SR Baseline model has been introduced in Section “Coupling foundation of SR and ZTA basic function model”. In the MFRA model, the multi-fault quick recovery and avoidance mechanism based on SR pre-deployment link ring backup is mainly applied, and the configuration of test objects is shown in Table 8. There are 4 security tests and 1 overhead tests: control plane message tampering, data plane loop attack, identity deception, and DoS attack. The simulation network topology is shown in Fig. 9, in which the components of the SR Baseline model and MFRA model are shown by the red box in the figure, that is, they include the SR native network composed of 5 Cisco xrv9k routers and 1 OpenDaylight controller based on KVM; ZbSR model, based on KVM, additionally set an information base, an expansion controller and a key center, and each router is connected with another KVM virtual machine as an agent. The control plane components and data plane topology of the SDN cross bitmap algorithm model and the DoS attack detection model based on C4.5, are consistent with the SR Baseline model, except that the data plane uses SDN switches.

**Table 8:** Test object configuration.

	ZbSR	Baseline	MFRA
Physical machine/virtual machine operating system	Ubuntu18.04/Ubuntu16.04		
Physical machine CPU	Core i7-7700 3.6 GHz		
Physical/virtual machine memory	32 GB/2 GB		
Controller	OpenDaylight		
Southbound interface	PCEP/NETCONF		
Data plane	MPLS		
Additional security mechanism	Data exchange security algorithm, network audit security algorithm	None	Backup, multi-failure recovery and re-failure avoidance

### Safety performance test and analysis

Due to the lack of comprehensive SR security model facing multiple threats, 4 models are introduced here, namely SR Baseline model, MFRA model, SDN cross bitmap algorithm model and DoS attack detection model based on C4.5, which are respectively compared with ZbSR model proposed in this paper in different types of attack tests. The threat model is set as follows: the attacker will implement 4 kinds of attack based on different switching devices and terminals, which one is the message tampering, namely the attacker tamper with the control plane message of a switching equipment, through the routing protocol flooding mechanism or other ways. This attack will induce the original flow path changes, to test whether the ZbSR model, the SR Baseline model, and the SDN cross bitmap algorithm model can prevent this attack. The second is the routing-loop attack, that is, by pressing the specified MPLS label stack into the head node of the traffic, the loop attack packet is constructed, so that the traffic transmission path will generate a loop, and whether the ZbSR model, the SR Baseline model can prevent the attack consequences is tested. The third is identity spoofing, that is, the Iperf tool is used to inject background traffic with a specified proportion of traffic characteristics, and part of the traffic is regarded as malicious traffic generated by identity spoofing, then the precision rate and recall rate (the probability of malicious traffic being detected and identified) of the ZbSR model, the SR Baseline model, and the MFRA model are tested. Fourth, DoS attack, that is, according to the simulated network set in Fig. 9, the packet generation rate threshold in the network audit security algorithm is set as 10,000/s, and the hping3 3.0.0 tool deployed on host H1 launches a DoS attack on the data plane with a packet sending rate of about 14,000/s, to test whether the ZbSR model, the SR Baseline model, the DoS attack detection model based on C4.5 and the MFRA model can effectively deal with DoS attack. In order to better evaluate the safety performance of each model, the precision rate and recall rate indexes are defined according to Table 9 as shown in the formula.

10 $$ precision = \frac{TP}{{TP + FP}} $$

11 $$ recall = \frac{TP}{{TP + FN}} $$

**Table 9:** Reference table of indicators.

Actual label	Predicted results	
	Malicious	Benign
Malicious	*TP*	*FN*
Benign	*FP*	*TN*

#### Message tampering

**Figure 10:**
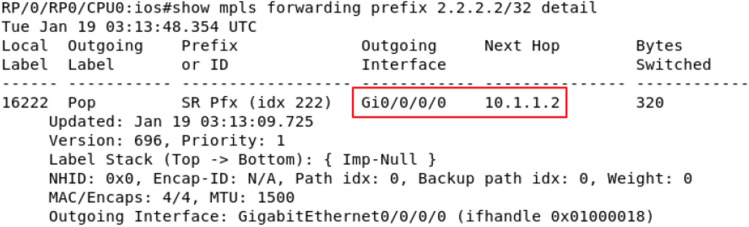
Original R1 device packet.

**Figure 11:**
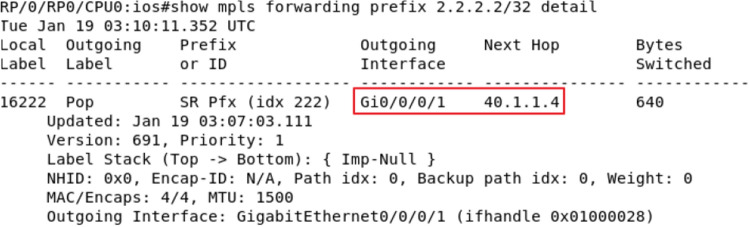
R1 device packet after tampering.

Configure the Loopback 0 address of the 2.2.2.2/32 for the R2 device, and assign it SID: 16,222. At this time, it is found that the next-hop corresponding to the tag 16,222 in the source route on the R1 device points to R2 by grabbing the packet with the Wireshark tool, as shown in Fig. 10. At this time, the attacker tampered with the control plane message using routing protocol flooding mechanism, etc., set the Loopback 0 address of R4 device to the same 2.2.2.2/32 as R2 and set the link cost value between R1 and R4 to half of the link cost value between R1 and R2. At this time, because the SR Baseline model use none additional security mechanisms, the next-hop corresponding to R1 selection label 16,222 will prefer R4. As shown in Fig. 11, in this case, the traffic path has been tampered. However, According to the SID audit mechanism in the security audit algorithm, the ZbSR model finds that this tampering is a malicious operation and rejects the packet tampering, so the traffic path is still shown as Fig. 10. The cross-bitmap algorithm model can also resist the similar tampering attack. The ZbSR model and the SDN cross bitmap algorithm model, which can defend against packet tampering attacks, are repeatedly executed 100 times based on SR network and SDN network respectively. The detection accuracy of the two models are both higher than 97%, and there is no significant difference. However, the SDN cross bitmap algorithm model can only defend against tampering attacks that can cause flow rule conflicts, and its universality is limited.

#### Routing-loop attack

**Figure 12:**
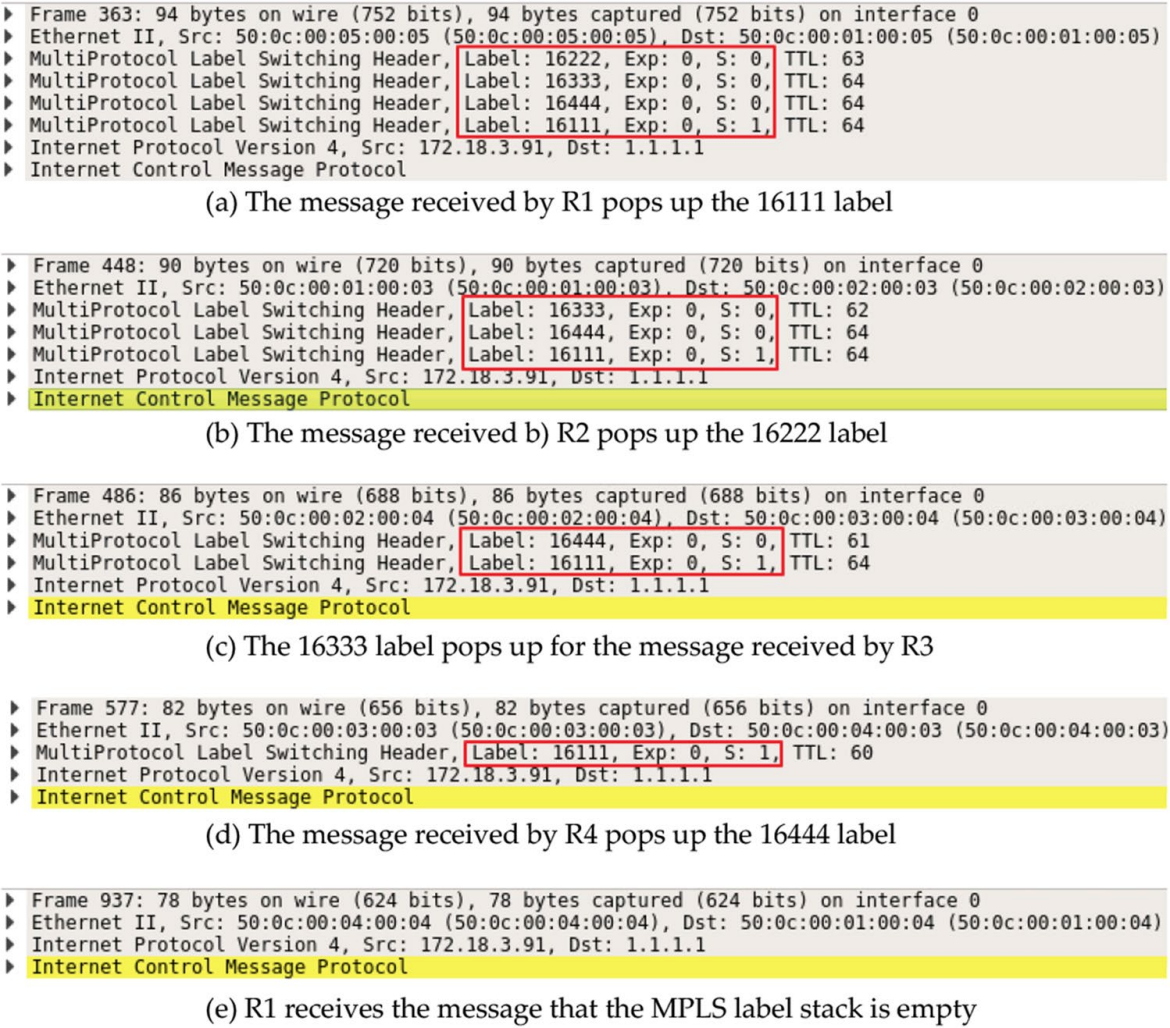
The process of jumping out MPLS labels from R1 to R4.

By pressing the MPLS label stack {R1 → R2 → R3 → R4 → R1} into R1 to construct a loop attack packet, and capturing packets from R1 ~ R4, the message flow of Fig. 12a–e can be obtained in the Baseline model. It can be seen that the stream starts from R1 (the source IP is 1.1.1.1), and the MPLS labels from R1 to R4 pop up hop by hop. However, the ZbSR model detects and discards the loop attack packets through the loop audit algorithm, and the above attack consequences do not occur.

#### Identity deception

**Figure 13:**
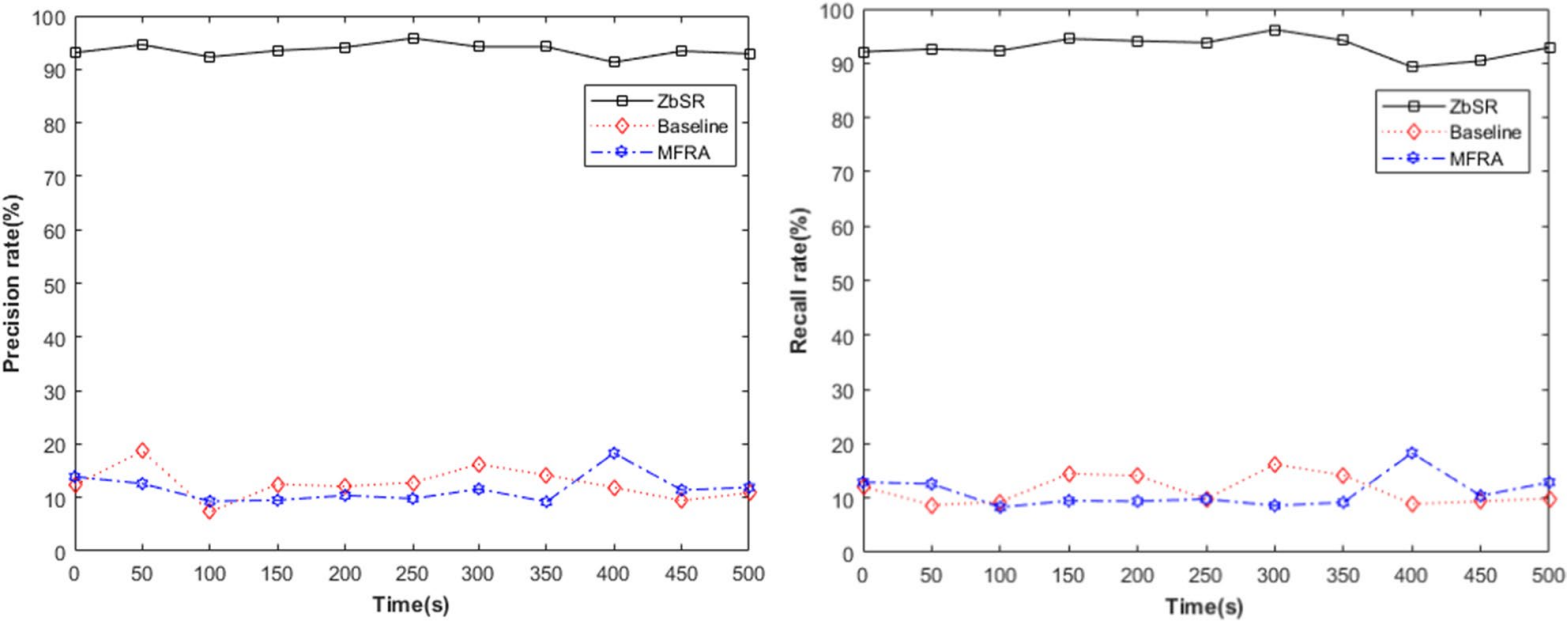
Precision rate and recall rate of malicious flow in 3 models.

To simulate the real network scene, the Iperf tool is used to inject the background traffic with the ratio of normal traffic to malicious traffic of 3: 1. Let the Smac and SID in the Credit information correspond to the mac and Prefix Node SID of R5, the pSID corresponds to the Prefix Node SID of R1, R2, and R3, the traffic with P as OSPF/ISIS is normal traffic, and the others are malicious traffic. The precision rate and recall rate of malicious traffic (the probability of malicious traffic being detected and identified) can be obtained by statistics, as shown in Fig. 13. It can be seen that based on traffic characteristics, the ZbSR model can perform identity authentication according to the *subalgorithm*_*IA*_ in Section “Data exchange security algorithm based on trust evaluation”, identify and prevent identity deception attacks with high accuracy, and ensure the credibility of the identity of both communication parties. In contrast, the Baseline model and MFRA model can filter a small amount of malicious traffic thanks to SR native security mechanism.

#### DoS attack

**Figure 14:**
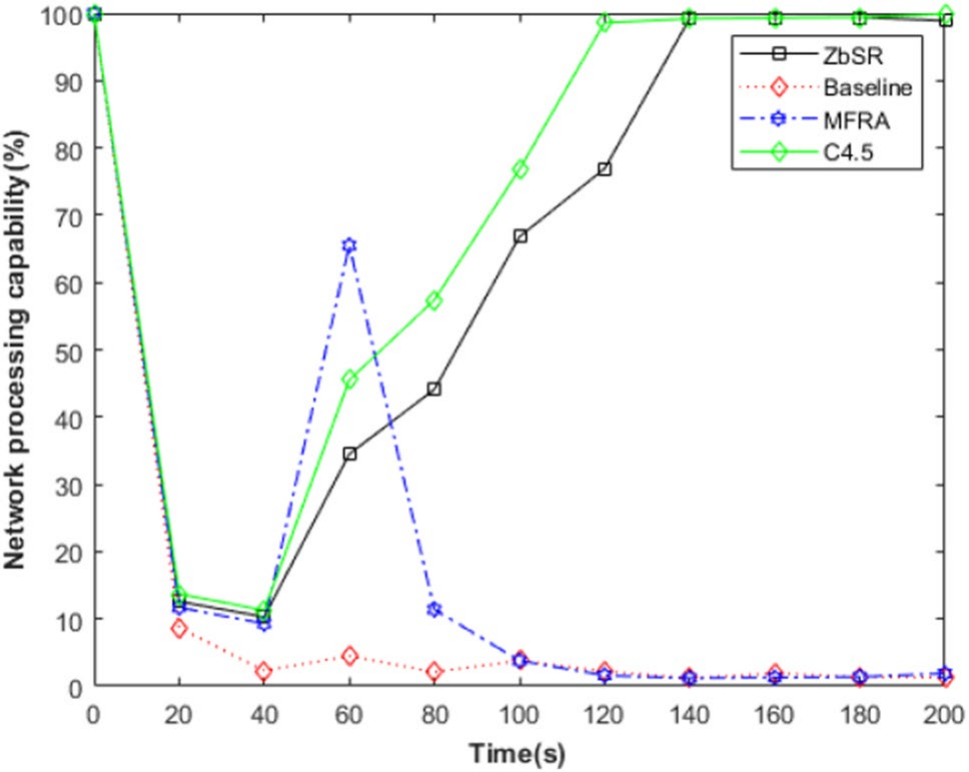
Comparison of the decline of the processing capacity of 4 models.

As shown in Fig. 14, the horizontal axis is the network running time, and the vertical axis is the network processing capacity, which is measured by the retention rate of the source route generation rate (this value is 1 when the network is normal). DoS attack started at 20 s. It can be seen that after the network processing capability based on the ZbSR model is temporarily degraded, the network can locate the injection node of malicious traffic through trust estimation and traffic auditing, and recover the processing capability gradually by filtering the attack traffic. The processing ability of the Baseline model drops rapidly after malicious traffic is injected; in the MFRA model, the network processing capacity is temporarily restored because the backup link is enabled after the congested link is detected, but the backup link also quickly becomes congested. The recovery speed of DoS attack detection model based on C4.5 is faster than that of ZbSR model, because the former adopts mature machine learning algorithm to detect attack traffic. However, this model is similar to the SDN cross bitmap algorithm model, because it cannot resist other types of attacks, and its universality is limited. In the ZbSR model, when the traffic auditing mechanism discovers traffic anomalies, multiple network nodes need to reduce the recommendation trust evaluation value of the malicious node to locate them accurately, so it is more time-consuming.

### Performance overhead test and analysis

**Figure 15:**
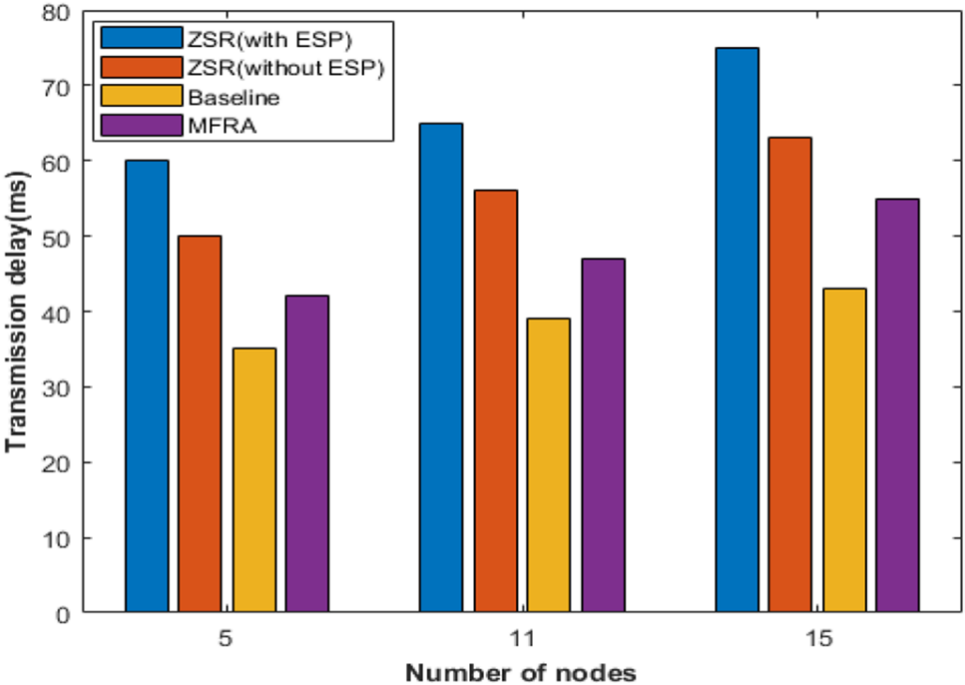
Comparison of streaming transmission delay of 3 models.

Since the C4.5 model and the SDN cross bitmap algorithm model are essentially based on SDN and both are implemented based on OpenFlow switch flow table, they are not comparable enough in delay cost testing. Therefore, the ZbSR model, SR Baseline model and MFRA model are analyzed in this paper. The streaming transmission delay of the 3 models in the network with 5, 11, and 15 nodes is shown in Fig. 15.

It can be seen that when software encryption is not turned on (ZbSR (unencrypted)), compared with the Baseline model and the MFRA model, the time delay of the ZbSR model increases by 18.2–22.7% (average incremental time delay is 19.3%) and 8.5–12.3% (average incremental time delay is 10.4%), respectively, and the proportion of time delay increased compared with MFRA model decreases with the number of nodes. After software encryption is turned on (ZbSR (encrypted)), the delay of the ZbSR model is further improved, which is 37.4–41.1% higher than that of encryption without it, which is consistent with the cost analysis in Section “ZbSR model security overhead”. Considering that if the application layer of the SR network terminal device has a mature encryption mechanism, there is no need to enable the terminal device encryption of the ZbSR model, and the Baseline model and MFRA model will also significantly increase the delay after encryption is turned on, so the security cost of the ZbSR model is regarded as 19.3% of the average incremental delay compared with the Baseline model when encryption is not turned on. To reduce the security overhead of the model, we can consider introducing particular data encryption components to replace software encryption in the terminal device; besides, the ZbSR model should be configured on-demand to focus on auditing the backbone network nodes with a large degree of nodes or large flow.

## Conclusion and future work

This article analyzes the untrustworthy security problems of network elements and PKI/CA in the zero-trust network environment of SR, and points out that it can be attributed to the untrustworthiness of SR data exchange and network audit functions, but there is no corresponding supporting security mechanism at present. Focusing on the application of ZTA in the SR-BE/TE network to improve its data plane security performance, this paper proposes a ZbSR data plane security model based on ZTA and the corresponding data exchange and network audit security algorithms. Through simulation test, the proposed model can provide various security protection for SR-BE/TE data plane, but also exposes its disadvantages of high-security cost. In the next step, we will focus on the hardware design of security components, the improvement of the trust evaluation algorithm for trust renewal, and the incremental network attack surface introduced by the model.

## Data Availability

The data and algorithms in our graphs and tables only come from the research process itself, without using public data sets or publishing unavailable data.
